# The Ethanol Extract from *Lonicera* *japonica* Thunb. Regresses Nonalcoholic Steatohepatitis in a Methionine- and Choline-Deficient Diet-Fed Animal Model

**DOI:** 10.3390/nu7105423

**Published:** 2015-10-21

**Authors:** Thing-Fong Tzeng, Yu-Cheng Tzeng, Yu-Jou Cheng, Shorong-Shii Liou, I-Min Liu

**Affiliations:** 1 Department of Pharmacy and Master Program, Tajen University, Pingtung 90741, Taiwan; d850084@yahoo.com.tw (T.-F.T.); azyl2032@gmail.com (Y.-J.C.); ssliou@tajen.edu.tw (S.-S.L.); 2 St. Dominic’s Catholic High School, Kaohsiung 80288, Taiwan; 2770727@yahoo.com.tw

**Keywords:** non-alcoholic steatohepatitis (NASH), *Lonicera japonica* Thunb, methionine, and choline-deficient diet (MCDD), Jun N-terminal kinase, extra cellular signal-regulated kinase 1/2

## Abstract

Nonalcoholic steatohepatitis (NASH) is characterized as fat accumulation in the hepatic tissue associated with various degrees of inflammation and progressive fibrosis. The potent anti-inflammatory and ethnopharmacological properties of *Lonicera japonica* Thunb. (Caprifoliaceae) make it an excellent source of novel medicinal targets for the treatment of NASH. The aim of the study was to investigate the effects of *L. japonica* ethanol extract (LJEE) on NASH in mice. C57BL/6J mice were fed with methionine-choline-deficient diet (MCDD) for eight weeks to promote the development of NASH. After development of the model, the mice were administered LJEE once daily via oral gavage at doses of 100, 200, or 300 mg/kg for another four weeks. Simultaneous treatments with LJEE (300 mg/kg/day) resulted in pronounced improvements in liver steatosis, ballooning degeneration, and inflammation. LJEE prevented MCDD-induced plasma level increases in aspartate aminotransferase and alanine aminotransferase. LJEE significantly reduced hepatic malondialdehyde level and ameliorated hepatic inflammation and fibrosis in MCDD-fed mice, which were associated with down-regulation of cytochrome P450 2E1 suppression of multiple proinflammatory and profibrotic genes. LJEE can prevent hepatic steatosis by reducing hepatic peroxisome acyl-CoA:diacylglycerol acyltransferase 2 expression, as well as by inducing proliferator-activated receptor α expression. In addition, the LJEE treatments caused significant reduction in the phosphorylated form of Jun N-terminal kinase along with an increase in the phosphorylated level of extra cellular signal-regulated kinase 1/2. Our study demonstrated the protective role of LJEE in ameliorating nutritional steatohepatitis.

## 1. Introduction

Nonalcoholic fatty liver disease (NAFLD) is a condition in which excessive fat accumulates in the liver of a patient who does not have a history of alcohol abuse [1]. NAFLD is classified into two categories: simple steatosis, in which only steatosis is observed, and nonalcoholic steatohepatitis (NASH), in which, in addition to steatosis, lobular inflammation and liver cell injury are observed. Although the underlying mechanisms of disease progression remain poorly understood, the “two-hit” hypothesis described the pathophysiology of NASH [2]. Steatosis (a primary insult) can sensitize the liver to secondary serious insults including reactive oxygen/nitrogen species, gut-derived endotoxins, pro-inflammatory cytokines like tumor necrosis factor-alpha (TNFα), resulting in NASH development [3]. NASH may develop into liver cirrhosis or hepatocellular carcinoma [4]. The available treatment options for NASH include weight loss, dietary, and lifestyle modifications, use of insulin sensitizing, and lipid lowering drugs [5]. Furthermore, combinations of these approaches have also been tried for management of NASH [5]. Since NASH is a multifactorial disease, single target based therapy has limited implications.

*Lonicera japonica* Thunb. (Caprifoliaceae), also known as Japanese honeysuckle, ‘‘Jin Yin Hua’’ or ‘‘Ren Dong’’, native in the East Asian, is recognized as edible and medicinal food [6]. The edible buds and flowers could be made into liquor and tea in the folk diet [6]. At the same time, it could be used as medicine, cosmetics, ornamental groundcover, and so on [6]. The constituents of this plant have been previously investigated and shown to contain iridoid glucosides and polyphenolic compounds [7]. The main polyphenolic components in *L. japonica* are chlorogenic acid, caffeic acid, flavoyadorinin-B, methyl chlorogenate, and protocatechuic acid, make great contribution to its special activities [7]. In current Chinese Pharmacopoeia [8], chlorogenic acid has been officially used as the indicator compound to characterize the quality of this herb. The modern pharmacological studies showed that *L.* *japonica* had wide pharmacological actions, such as antibacterial, anti-inflammatory, antiviral, antiendotoxin, antipyretic, and other activities [6]. Since 1995, *Lonicera japonica* has been listed in the Pharmacopoeia of the People’s Republic of China [9], and made in some preparations to treat chronic enteritis, pneumonia, acute tonsillitis, nephritis, acute mastitis, leptospirosis in clinic. *L. japonica* also has been employed extensively to prevent and treat some serious viral diseases of human and veterinary, such as SARS coronavirus, H1N1 (Swine) flu virus [10]. In Chinese Pharmacopoeia, only the flowers and flower buds have been officially used as the active parts to treat diseases [8].

Pharmacological studies have demonstrated that *L.* *japonica* has anti-fibrotic effect in rats with fibrosis induced by dimethylnitrosamine [11]. It has also been reported that an herbal formula consisting of *L.* *japonica* attenuates carbon tetrachloride-induced liver damage in rats [12]; hepatoprotective potential of this plant could be considered. However, the possibility that *L.* *japonica* could prove beneficial in ameliorating NASH has not been previously explored. The use of a diet deficient in essential amino acids such as methionine and choline is a well-accepted model for inducing NASH, which recapitulates many of the features of this disease in humans, including a histologic picture that mimics that seen in human fibrotic disorders associated with hepatic lipid accumulation, and the presence of inflammation and oxidative stress [13]. To support the application of *L.* *japonica* in the treatment of NASH-like disorders, the study investigated the hepatotherapeutic effect of *L.* *japonica* in an animal model of methionine- and choline-deficient diet (MCDD)-induced hepatic injury and to elucidate the underlying mechanism involved.

Ciprofibrate, a fibric acid derivative, is a commercially available drug in the treatment of hyperlipidemia [14]. It has been documented that ciprofibrate at the daily oral dosage of 10 mg/kg was effective to ameliorate hyperlipidemia in hypertriglyceridemic mice [14]. In the present study, ciprofibrate was thus used as positive control to compare the effects of *L.* *japonica* on the amelioration of NASH.

## 2. Materials and Methods

### 2.1. Plant Material and Extraction

The flowering aerial parts of *L.* *japonica* were collected from Ligang Township (Pingtung County, Taiwan) in November 2014. Macroscopic and microscopic examinations, thin-layer chromatography, and high-performance liquid chromatography (HPLC) were used to confirm the authenticity of the plant material provided (this analysis was performed by Dr. Tang-Yao Hong, Department of Biotechnology, Collage of Pharmacy and Health Care, Tajen University). The voucher specimen (Lot No. LJ 20141121) has been preserved in our laboratory for future reference. The flowering aerial parts of *L.* *japonica* were air-dried, pulverized to a coarse powder in a mechanical grinder, and passed through a 40-mesh sieve to get powdered samples. Powdered samples (5 kg) were extracted at room temperature thrice with 10 L of 95% ethanol for 48 h on an orbital shaker to make the ethanol extracts. The *L. japonica* ethanol extract (LJEE) was evaporated to dryness under reduced pressure to completely eliminate alcohol and lyophilized, yielding approximately 582 g of dry residue (w/w yield: 11.3%). LJEE was stored at −20 °C until use and was suspended in distilled water. The content of chlorogenic acid in the samples was then analyzed using the Agilent 1100 HPLC system (Agilent Technologies, Inc., Santa Clara, CA, USA). The calibration equation of peak area against the concentration of chlorogenic acid was y = 6832.3× + 117.2 (*R*^2^ = 0.9998). The chlorogenic acid content in LJEE was 68.2 ± 0.3 μg/g of the dry extract.

### 2.2. Animal and Experimental Protocols

Fourty-eight six-week-old male C57BL/6 mice were obtained from the National Laboratory Animal Center (Taipei, Taiwan). They were maintained in a temperature-controlled room (25 ± 1 °C) on a 12h:12h light-dark cycle (lights on at 06:00 h) in our animal center. Food and water were provided ad libitum. The MCDD and the methionine- and choline-sufficient diet (MCSD) were purchased from Dyets Inc. (#518810 and #518754, respectively; Bethlehem, Pennsylvania, PA, USA). Both diets contained similar nutrients (14.2% protein, 15% fat, 3.09% ash, and 5% fiber), except that methionine and choline were not included in the MCDD, whereas 1.70 g/kg methionine and 14.48 g/kg choline bitartrate were provided in the MCSD. All animal procedures were performed according to the Guide for the Care and Use of Laboratory Animals of the National Institutes of Health, as well as the guidelines of the Animal Welfare Act. These studies were conducted with the approval of the Institutional Animal Care and Use Committee (IACUC) at Tajen University (IACUC 103-14, 12 December 2014).

After consuming a MCDD for eight weeks, a group of eight mice was dosed by oral gavage once per day either with LJEE doses of 100, 200, or 300 mg/kg in a volume of 1.5 mL/kg distilled water. The dosage regime was selected based on a previous report demonstrating that LJEE at 100 and 200 mg/kg was potentially effective in improving renal function in diabetic rats [15]. Another group of MCDD-fed mice (*n* = 8) was orally administered ciprofibrate (10 mg/kg/day) dissolved in distilled water, a dose based on a study that indicated that long-term treatment ameliorated hyperlipidemia in hypertriglyceridemic mice [14]. Vehicle-treated mice received 1.5 mL/kg distilled water only. A vehicle-treated group of MCDD-fed mice (*n* = 8) and a group of MCSD-fed mice (*n* = 8) were treated with 1.5 ml/kg distilled water only over the same treatment period.

Following the further four-week treatment, animals were weighed, fasted for 12 h, and sacrificed under intraperitoneal injection of a mixture of ketamine (100 mg/kg) and xylazine (20 mg/kg). Blood samples from the inferior vena cava were collected into heparinized syringe (15 units/mL blood). The left lobe of the liver was removed, rinsed with physiological saline, and stored immediately at −80 °C in liquid nitrogen until assayed. The right lobe of the liver was fixed in 10% neutralized formalin for histology. Relative liver weight was the liver weight divided by body weight.

### 2.3. Biochemical Analysis

Blood samples were centrifuged at 2000× *g* for 10 min at 4 °C. The plasma was removed and placed into aliquots for analyses. Diagnostic kits for determining plasma levels of total cholesterol (TC; Cat. # 10007640) and triglycerides (TG; Cat. # 10010303) were purchased from Cayman Chemical Company (Ann Arbor, MI, USA). Kits for determination of plasma alanine aminotransferase (ALT; EC 2.6.1.2; Cat. No. A524-780TM) and aspartate aminotransferase (AST; EC 2.6.1.1; Cat. No. A559-780TM) concentrations were purchased from Teco Diagnostics (Anaheim, CA, USA). All experimental assays were carried out according to the manufacturers’ instruction; all samples were analyzed in triplicate.

### 2.4. Measurement of Hepatic Lipids

Hepatic lipid content was determined from fresh liver samples. Liver (1.25 g) was homogenized with chloroform/methanol (1:2, 3.75 mL) and mixed well with chloroform (1.25 mL) and distilled water (1.25 mL). After centrifuging for 10 min at 1500× *g*, the lower clear organic phase was transferred into a new glass tube and then lyophilized. The lyophilized powder was dissolved in chloroform/methanol (1:2) and stored at −20 °C for less than three days [16]. Hepatic cholesterol and TG levels in the lipid extracts were analyzed using the same diagnostic kits used for plasma analysis.

### 2.5. Lipid Peroxidation Assay

The liver malondialdehyde (MDA) levels, as an index of lipid peroxidation, were determined by the double heating method [17]. The method is based on spectrophotometric measurement of the purple color generated by the reaction of thiobarbituricacid (TBA) with MDA. Each liver tissue was homogenized (10% w/v) in 25 mmol/L Tris–HCl pH7.4, and centrifuged at 10,000 rpm for 15 min. The supernatant aliquots were stored at −70 °C until assay. For MDA assay, 2.5 mL of trichloroacetic acid solution (10%) was added to 0.5 mL of the supernatant followed by heating in a boiling water bath for 15 min. After cooling to room temperature, the samples were centrifuged at 3000 rpm for 10 min, and then 2 mL of the reaction mixture was transferred to a test tube containing 1 mL of TBA solution (0.67%). Each tube was then placed in a boiling water bath for 15 min. After cooling to room temperature, the absorbance was measured at 532 nm. The concentration of MDA was calculated based on the absorbance coefficient of the TBA-MDA complex.

### 2.6. Histological Analysis

For the microscopic analysis, the liver fragment slides were stained with hematoxylin and eosin (H&E) and subsequently assessed by a single pathologist who was unaware of the experimental groups. The histopathological features of steatohepatitis were evaluated semi-quantitatively, according to the validated histological scoring system recommended by the Pathology Committee of the NASH Clinical Research Network as follows: steatosis grade (0–3; 0:<5%, 1:5%–33%, 2: 33%–66%, and 3:>66%), lobular inflammation (0–3; 0:no foci, 1: <2 foci per 200× field, 2: 2–4 foci per 200× field, and 3: >4 foci per 200× field), hepatocyte ballooning (0–2; 0:None, 1:few balloon cells, and 2:many cells/prominent ballooning) [18]. All measurements were performed under a light microscope with an attached digital camera (C3040-AD6, Olympus, Tokyo, Japan) by an experienced pathologist.

### 2.7. Western Blotting

Liver samples were homogenized for 30 min in ice-cold radioimmune protection assay lysis buffer (50 mmol/L Tris, pH 7.4, 150 mmol/L NaCl, 1% Triton X-100, 1% sodium deoxycholate, 0.1% sodium dodecyl sulfate, 1 mg/mL leupeptin, 50 mmol/L sodium fluoride, 1 mmol/L sodium orthovanadate, and 1 mmol/L phenylmethylsulfonyl fluoride). Liver homogenates were then centrifuged at 12,000 rpm for 30 min at 4 °C and protein concentrations were determined using the Bradford method. Equal protein amounts (50 μg) were separated by sodium dodecyl sulfate polyacrylamide gel electrophoresis and electro-transferred onto polyvinylidene difluoride membranes, which were then blocked with 1% bovine serum albumin and probed with primary antibodies against extra cellular signal-regulated kinase 1/2 (ERK 1/2; Santa Cruz Biotechnology, Santa Cruz, CA, USA), phospho-ERK 1/2 (Thr202/Tyr204) (Santa Cruz, CA, USA), Jun N-terminal kinase (JNK; Santa Cruz Biotechnology), phospho-JNK (Thr183/Tyr185) (Santa Cruz Biotechnology), p38 mitogen-activated protein kinase (p38; Cell Signaling Technology, Beverly, MA, USA), phospho-p38 (Thr180/Tyr182) (p-p38; Cell Signaling Technology), or β-actin (Santa Cruz Biotechnology). The level of β-actin was estimated for equal loading of sample. Membranes were washed three times with Tris-buffered saline Tween 20 (TBST) and incubated for 1 h at room temperature with appropriate horseradish peroxidase-conjugated secondary antibodies. After three additional TBST washes, the immunoreactive bands were visualized by enhanced chemiluminescence (Amersham Biosciences, Buckinghamshire, UK) according to the manufacturer's instructions. Band densities were determined using ATTO Densitograph Software (ATTO Corporation, Tokyo, Japan). In all experiments, equal protein loadings have been confirmed by the β-actin content.

### 2.8. Analysis of mRNA Expression of Hepatic Genes

To analyze gene expression, total RNA was extracted from 100-mg frozen liver samples using Trizol reagent (Invitrogen; Boston, MA, USA). RNA was quantified by A260, and its integrity verified by agarose gel electrophoresis using ethidium bromide for visualization. For the reverse transcriptase reaction, 1 μg of total RNA per sample and 8.5 μg/μL random hexamer primers were heated to 65 °C for 5 min, and then quenched on ice. This mixture was combined with 500 μmol/L each of dATP, dTTP, dCTP, and dGTP, 10 mmol/L DTT, 20 mmol/L Tris-HCl (pH 8.4), 50 mmol/L KCl, 5 mmol/L MgCl_2_, 40 units of RNaseOUT recombinant ribonuclease inhibitor (Invitrogen), and 100 units SuperScript III reverse transcriptase (Invitrogen). Samples were treated with DNase (Promega; Madison, WI, USA) for 20 min at 37 °C in a GeneAmp 9700 Thermal Cycler (Applied Biosystems; Foster City, California, CA, USA) and then held at 4 °C. After aliquots were taken for immediate use in polymerase chain reaction (PCR), the remainder of the cDNA was stored at −20 °C. Messenger RNA (mRNA) expression was measured by quantitative real-time reverse transcription polymerase chain reaction (RT-PCR) in a fluorescent temperature Lightcycler 480 (Roche Diagnostics; Mannheim, Germany). Primers for amplification of each gene are listed in Table 1. The highly specific measurement of mRNA was carried out for cytochrome P450 (CYP) 2E1, tumor necrosis factor (TNF)α, transforming growth factor (TGF)-β, α-smooth muscle actin (SMA), type I collagen (collagen I), matrix metalloproteinase (MMP)2 and MMP9, peroxisome acyl-CoA:diacylglycerol acyltransferase 2 (DGAT2), proliferator-activated receptor (PPAR) α, and β-actin using the LightCycler system (Bio-Rad). Each sample was run and analyzed in duplicate. Primers were designed using Primer Express Software version 2.0 System (Applied Biosystems; Foster City, CA, USA). The PCR reaction was performed using the following cycling protocol: 95 °C for 5 min, 45 cycles of 95 °C for 5 s, 58 °C for 15 s, and 72 °C for 20 s. Dissociation curves were run after amplification to identify the specific PCR products. All mRNA levels were normalized to β-actin mRNA values and the results expressed as fold changes of the threshold cycle (Ct) value relative to controls using the delta-delta Ct method [19]. To ensure amplification specificity during RT-PCR, amplified products were subjected to agarose gel electrophoresis to visually confirm the presence of a single amplicon of the expected size.

**Table 1:** Sequences of primers used for real-time RT-PCR analysis in this study.

Target Gene	Primers	Sequence
CYP2E1	FP	ATGTCATCCCCAAGGGTACA
	RP	CGGGGAATGACACAGAGTTT
TNFα	FP	CCAGGAGAAAGTCAGCCTCCT
	RP	TCATACCAGGGCTTGAGCTCA
TGF-β	FP	CCCAGCATCTGCAAAGCTC
	RP	GTCAATGTACAGCTGCCGCA
α-SMA	FP	TGCTGTCCCTCTATGCCTCT
	RP	GAAGGAATAGCCACGTCAG
Collagen I	FP	ACAGCCGCTTCACCTACAGC
	RP	TCAATCACTGTCTTGCCCCA
MMP2	FP	AACTTTGAGAAGGATGGCAAGT
	RP	TGCCACCCATGGTAAACAA
MMP9	FP	CCCCAAAACGGACAAAGAG
	RP	CTTCAGCACAAAACGGTTGC
DGAT2	FP	CATGAAGACCCTCATCGCCG
	RP	GTGACAGAGAAGATGTCTTGG
PPARα	FP	CGTCCTGGCCTTCTAAACGTAG
	RP	CCTGTAGATCTCCTGCAGTAGCG
β-actin	FP	TGTGATGGTGGGAATGGGTCAG
	RP	TTTGATGTCACGCACGAT TTCC

FP, forward primer; RP, reverse primer.

### 2.9. Statistical Analysis

Data are expressed as mean ± standard deviation (SD). Statistical analyses were performed using one-way analysis of variance. Dunnett range post-hoc comparisons were used to determine the source of significant differences, where appropriate. The SigmaPlot (Version 12.0) (Systat Software, San Jose, CA) program was used for statistical analysis. Values of *p* < 0.05 were considered statistically significant.

## 3. Results

### 3.1. Effects on Body Weights and Liver Weights in Mice

A MCDD remarkably decreased mouse body and liver weights as compared with the MCSD (Table 2). Treating MCDD-fed mice with LJEE (300 mg/kg/day) did not result in any significant effects on body weight, absolute liver weight, and the relative liver weight (Table 2). Similar results were found for MCDD-fed mice that were treated with ciprofibrate (Table 2).

### 3.2. Effects of Treatments on Plasma and Hepatic Lipids in Mice

Plasma levels of TC, and TG were lower in MCDD-fed mice than that in MCSD-fed animals. Treatment of MCDD-fed mice with LJEE or ciprofibrate had no significant effect on plasma TC and TG (Table 2).

Hepatic cholesterol and TG levels were significantly higher in MCDD-fed mice compared with mice from the MCSD-fed group. Treatment MCDD-fed mice with LJEE (300 mg/kg/day) reduced the hepatic cholesterol and TG levels by 30.9% and 29.2%, respectively, compared to the levels in vehicle-treated MCDD-fed mice (Table 2). The hepatic levels of cholesterol and TG in MCDD-fed mice receiving ciprofibrate treatment were decreased by 33.6% and 40.5%, respectively, compared to those in vehicle-treated MCDD-fed mice (Table 2).

### 3.3. Effects of Treatments on Liver Injury in Mice

Plasma ALT and AST activities in MCDD-fed mice were higher than those in MCSD-fed group (Table 2). The plasma ALT and AST activities were markedly reduced in MCDD-fed mice treated for four weeks with 300 mg/kg/day LJEE (Table 2). Ciprofibrate treatment also significantly attenuated the changes in plasma ALT and AST activities in MCD diet-fed mice (Table 2).

**Figure 1:**
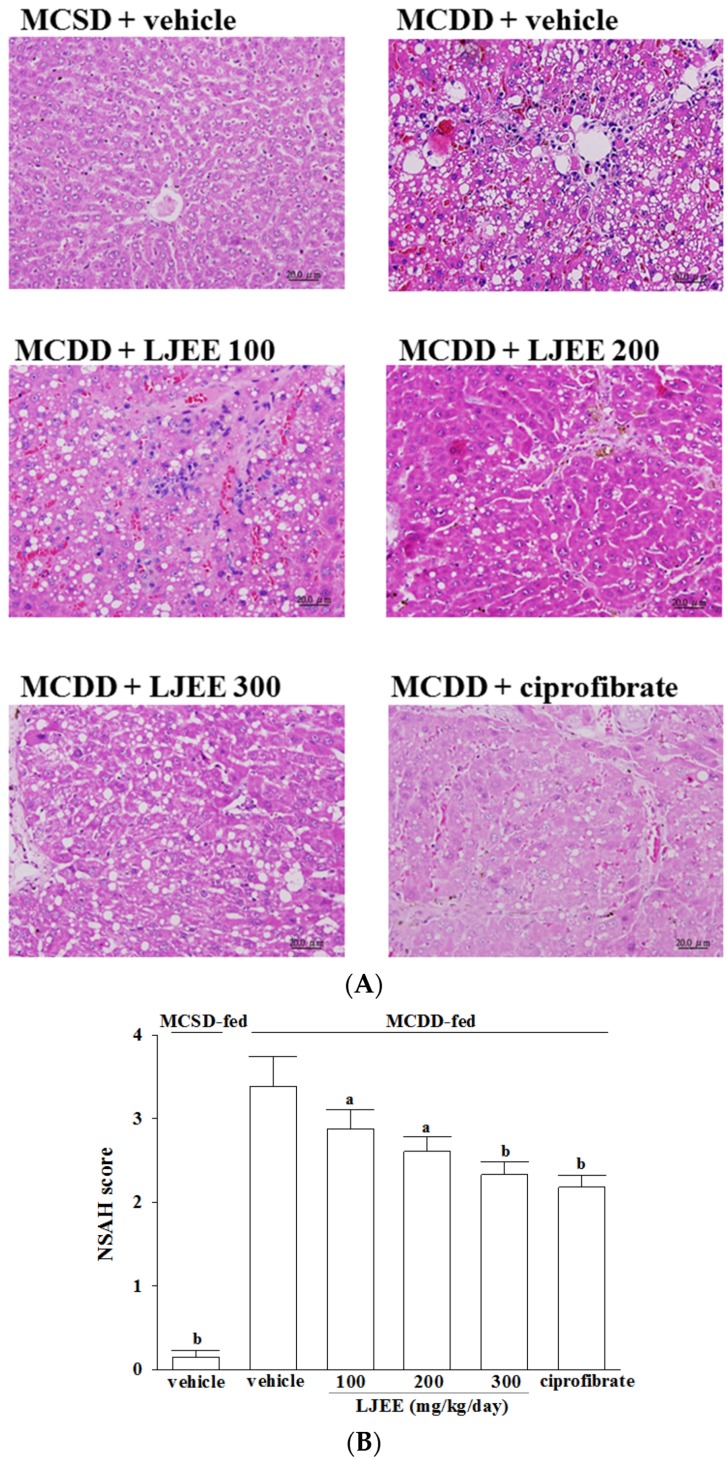
Effects of treatments on liver histology. (**A**) Representative images of hematoxylin and eosin stained livers from methionine- and choline-sufficient diet (MCSD)- and methionine-choline-deficient diet (MCDD)-fed mice after four weeks of LJEE or ciprofibrate treatment. The vehicle (distilled water) used to prepare the test medication solutions was given at the same volume. Photomicrographs (original magnification, 200×) of livers isolated from vehicle-treated MCSD-fed mice (MCSD + vehicle), vehicle-treated MCDD-fed mice (MCDD + vehicle), 100 mg/kg/day (MCDD + *L. japonica* ethanol extract (LJEE) 100), 200 mg/kg/day (MCDD + LJEE 200), and 300 mg/kg/day (MCDD + LJEE 300) LJEE-treated MCDD-fed mice. Another group of MCDD-fed mice was orally administered 10 mg/kg/day of ciprofibrate (MCDD + ciprofibrate); (**B**) Sections were evaluated in a blinded manner by pathologists, and a scoring method was assigned as described in experimental methods for NASH evaluation. Values (mean ± standard deviation (SD)) were obtained from each group of eight animals. ^a^ *p* < 0.05 and ^b^ *p* < 0.01 compared to the values of vehicle-treated MCDD-fed mice in each group, respectively.

Liver sections from the MCSD-fed group stained with H&E showed normal hepatic cells and histoarchitecture (Figure 1A). The MCDD induced measurable hepatic steatosis (mainly with a predominant macrovesicular pattern) in mice and this progressed to inflammation and fibrosis (Figure 1A). Hepatic steatosis, lobular inflammation, and hepatocyte ballooning were significantly reduced in the LJEE (300 mg/kg/day) and ciprofibrate-trated group compared to the MCDD-only fed group (Figure 1A). Liver histology was evaluated using the NASH score (Figure 1B).

**Table 2:** Effects on blood biochemistry and hepatic parameters in MCSD- or MCDD-fed mice receiving four weeks of treatments.

	MCSD	MCDD				
	Vehicle	Vehicle	LJEE (mg/kg/day)			Ciprofibrate
			100	200	300	(10 mg/kg/day)
Initial body weight (BW) (g)	20.71 ± 1.51	20.67 ± 1.25	20.75± 1.46	20.61 ± 1.33	20.72 ± 1.57	20.78 ± 1.75
Final BW (g)	28.04 ± 1.72 ^b^	14.64 ± 1.48	14.94 ± 1.59	15.23 ± 1.50	14.98 ± 1.64	15.16 ± 1.76
Liver absolute weight (g)	1.44 ± 0.11 ^b^	0.51 ± 0.07	0.53 ± 0.09	0.57 ± 0.08	0.57 ± 0.06	0.56 ± 0.05
Liver relative weight (%)	5.13 ± 0.23 ^a^	3.48 ± 0.28	3.54 ± 0.31	3.74 ± 0.28	3.80 ± 0.29	3.71 ± 0.32
Plasma TC (mg/dL)	150.82 ± 4.07 ^b^	24.88 ± 3.91	23.87 ± 4.13	23.77 ± 3.26	25.13 ± 3.83	25.96 ± 2.96
Plasma TG (mg/dL)	95.84 ± 4.26 ^b^	50.78 ± 3.83	55.79 ± 3.46	52.29 ± 3.57	50.91 ± 4.11	54.32 ± 3.62
Plasma ALT (U/L)	40.26 ± 7.13 ^b^	303.08 ± 16.23	287.85 ± 17.26 ^a^	253.37 ± 15.78 ^a^	184.05 ± 14.56 ^b^	126.94 ± 17.42 ^b^
Plasma AST (U/L)	103.69 ± 9.83 ^b^	441.62 ± 18.26	393.24 ± 17.34 ^a^	282.46 ± 19.33 ^a^	224.63 ± 18.14 ^b^	188.74 ± 15.36 ^b^
Hepatic TC (µmol/g liver)	11.61 ± 0.83 ^b^	21.61 ± 1.24	18.44 ± 1.13 ^a^	16.67 ± 1.21^a^	14.92 ± 1.09 ^b^	14.27 ± 1.16 ^b^
Hepatic TG (µmol/g liver)	8.72 ± 0.73 ^b^	17.68 ± 1.14	14.74 ± 1.26	12.52 ± 1.38 ^a^	11.18 ± 1.12 ^a^	10.51 ± 1.07 ^b^
Hepatic MDA (µmol/g liver)	10.23 ± 2.31 ^b^	393.14 ± 15.26	334.16 ± 16.17 ^a^	314.52 ± 13.27 ^a^	294.85 ± 17.13 ^b^	282.96 ± 16.21^b^

The vehicle (distilled water) used to prepare the tested medication solution was given at the same volume. Values (mean ± SD) were obtained from each group of eight animals in each group after four weeks of the experimental period. ^a^ *p* < 0.05 and ^b^ *p* < 0.01 compared to the values of vehicle-treated MCDD-fed mice in each group, respectively.

### 3.4. Effects of Treatments on Oxidative Stress in Livers of Mice

The hepatic level of MDA in MCDD-fed mice was markedly higher than that in the MCSD-fed group (Table 2). LJEE treatment attenuated the hepatic MDA levels of MCDD-fed mice in a dose-dependent manner (Table 2). Hepatic MDA levels in MCDD-fed mice were also decreased by ciprofibrate treatment (Table 2).

### 3.5. Effects of Treatments on Hepatic mRNA Expression of NASH-Related Specific Genes in Mice

Hepatic mRNA expression levels of CYP2E1, TNFα, and TGF-β1 in the MCDD-fed mice were increased by 2.7-, 3.2-, and 2.3-fold, respectively, compared to those in the MCSD-fed group (Table 3). However, treatment MCDD-fed mice with LJEE (300 mg/kg/day) reduced the hepatic mRNA expression of CYP2E1 (0.6-fold), TNFα (0.7-fold), and TGF-β1 (0.7-fold) relative to expression levels in vehicle-treated MCDD-fed mice (Table 3). Treatment MCDD-fed mice with ciprofibrate reduced the hepatic mRNA expression of CYP2E1 (0.7-fold), TNFα (0.7-fold), and TGF-β1 (0.6-fold) relative to those in vehicle-treated MCDD-fed mice (Table 3).

Compared with MCSD-fed group, the hepatic mRNA levels of α-SMA, collagen I, MMP2, and MMP9 in MCDD-fed mice increased obviously (3.1-, 3.3-, 2.7-, and 3.2-fold, respectively; Table 3), which were down-regulated by LJEE (300 mg/kg/day) treatment (30.8, 38.3, 25.8, and 34.2% decreases, respectively; Table 3). The hepatic mRNA levels of α-SMA, collagen I, MMP2, and MMP9 in MCDD-fed mice receiving ciprofibrate treatment were decreased to 71.8, 59.2, 70.5, and 60.8% relative to the expression levels in vehicle-treated counterparts, respectively (Table 3).

Hepatic mRNA levels of DGAT2 were significantly reduced (by 51.7%) in LJEE (300 mg/kg/day)-treated MCDD-fed mice compared with the vehicle-treated counterparts (Table 3). Ciprofibrate suppressed the MCDD-induced stimulation in hepatic mRNA levels of DGAT2 to 58.6% relative to those in their vehicle-treated counterparts (Table 3).

The mRNA levels of PPARα in livers of MCDD-fed mice were lower to 47.6% of those from MSCD-fed group (Table 3). The hepatic mRNA levels of PPARα were upregulated by LJEE (300 mg/kg/day) or ciprofibrate treatment, with an increase of 57.4, and 65.9%, when compared with those observed in the vehicle-treated counterparts (Table 3).

### 3.6. Effects of Treatments on MAPK Signaling Pathways in Mice

**Figure 2:**
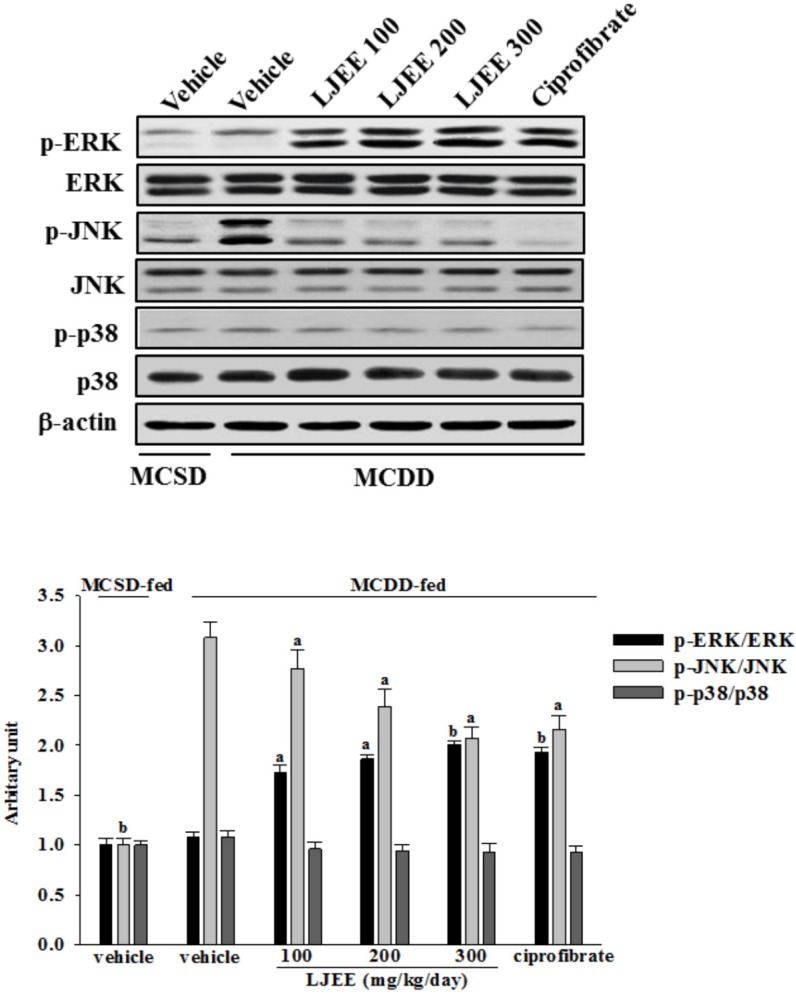
Effects of treatments on mitogen-activated protein kinase (MAPK) signaling pathways in livers of mice. A representative western blot pattern of hepatic MAPK activation from MCSD- and MCDD-fed mice after four weeks of LJEE or ciprofibrate treatment. MCDD-fed mice were dosed by oral gavage once daily for four weeks with LJEE at 100 (LJEE 100), 200 (LJEE 200) or 300 mg/kg/day (LJEE 300). Another group of MCDD-fed mice was orally administered 10 mg/kg/day of ciprofibrate (ciprofibrate). MCSD- or MCDD-fed mice receiving vehicle treatment were given the same volume of vehicle (distilled water) used to prepare the tested medication solutions. Ratios of p-ERK/ERK, p-JNK/JNK, or p-p38/p38 expressed as the mean with SD (*n* = 8 per group) in each column have been shown in the lower panel. ^a^ *p* < 0.05 and ^b^ *p* < 0.01 compared to the values of vehicle-treated MCDD-fed mice in each group, respectively.

No considerable alteration was observed in the phosphorylated form of ERK among MCDD-fed mice relative to MCSD-fed group. Treatment MCDD-fed mice with the LJEE resulted in a significant increase in the extent of ERK phosphorylation (Figure 2). Similar results were obtained in the ciprofibrate-treated MCDD-fed group (Figure 2).

The JNK phosphorylation was significantly greater in the livers of MCDD-fed mice compared to the MCSD-fed group (Figure 2). LJEE (300 mg/kg/day)-treated MCDD-fed mice had 32.8% lower JNK phosphorylation in liver than that of their vehicle-treated counterparts (Figure 2). Ciprofibrate suppressed the hepatic JNK phosphorylation of MCDD-fed mice by 29.8% relative to that of their vehicle-treated counterparts (Figure 2).

No change was observed in the phosphorylation of p38 in the livers of MCDD-fed mice compared with the MCSD-fed group (Figure 2). Treatment MCDD-fed mice with LJEE or ciprofibrate did not affect the phosphorylation of p38 in the livers (Figure 2).

No alteration in total hepatic ERK, JNK, and/or p38 contents has occurred among different groups (Figure 2).

**Table 3:** Effects on hepatic mRNA expression of NASH-related specific genes in MCSD- or MCDD-fed mice receiving four weeks of treatments.

Relative Expression	MCSD	MCDD				
	Vehicle	Vehicle	LJEE (mg/kg/day)			Ciprofibrate
			100	200	300	10 mg/kg/day
CYP2E1 mRNA	1.00 ± 0.02 ^b^	2.71 ± 0.23	2.43 ± 0.26	2.18 ± 0.13 ^a^	1.82 ± 0.16 ^b^	1.94 ± 0.11 ^b^
TNF-α mRNA	1.00 ± 0.05 ^b^	3.15 ± 0.23	2.81 ± 0.28	2.63 ± 0.19 ^a^	2.31 ± 0.17 ^a^	2.26 ± 0.15 ^b^
TGF-β mRNA	1.00 ± 0.04 ^b^	2.34 ± 0.13	1.93 ± 0.09	1.86 ± 0.07 ^a^	1.73 ± 0.09^a^	1.56 ± 0.08 ^a^
α-SMA mRNA	1.00 ± 0.06 ^b^	3.08 ± 0.19	2.77 ± 0.08	2.46 ± 0.11 ^a^	2.13 ± 0.09 ^b^	2.21 ± 0.13 ^b^
Collagen I	1.00 ± 0.05 ^b^	3.26 ± 0.17	3.04 ± 0.12	2.53 ± 0.08 ^a^	2.02 ± 0.09 ^b^	1.93 ± 0.12 ^b^
MMP2	1.00 ± 0.06 ^b^	2.71 ± 0.16	2.43 ± 0.15	2.16 ± 0.09 ^a^	2.01 ± 0.08 ^b^	1.91 ± 0.14 ^b^
MMP9	1.00 ± 0.05 ^b^	3.22 ± 0.26	2.89 ± 0.22	2.31 ± 0.21 ^a^	2.12 ± 0.18 ^b^	1.96 ± 0.11 ^b^
DGAT-2	1.00 ± 0.05 ^b^	0.87 ± 0.06	0.74 ± 0.05^a^	0.63 ± 0.04 ^a^	0.42 ± 0.04 ^a^	0.51 ± 0.03 ^a^
PPARα	1.00 ± 0.04 ^b^	0.47 ± 0.04	0.52 ± 0.06	0.62 ± 0.05 ^a^	0.74 ± 0.05 ^a^	0.78 ± 0.03 ^a^

The vehicle (distilled water) used to prepare the tested medication solution was given at the same volume. Values (mean ± SD) were obtained from each group of eight animals in each group after four weeks of the experimental period. ^a^ *p* < 0.05 and ^b^ *p* < 0.01 compared to the values of vehicle-treated MCDD-fed mice in each group, respectively.

## 4. Discussion

The histopathological features of NASH include evidence of steatosis, liver cell injury, a mixed inflammatory lobular infiltrate, and variable degrees of fibrosis [2]. The MCDD is a well-established and widely-used nutritional model of steatohepatitis that causes hepatic steatosis and inflammation that mimics NAFLD in humans [13]. In this study, mice consistently developed steatosis, ballooning injury, and inflammatory cell infiltration following the MCDD intake, which is in line with previous reports on this nutritional model of NASH [20]. The histopathological findings in liver of MCDD fed-mice were considerably ameliorated by LJEE treatment. We also found that the ALT and AST activities were markedly reduced in MCDD fed-mice treated with LJEE. AST and ALT are considered to be sensitive indicators of hepatocellular damage and within limits can provide a quantitative evaluation of the degree of damage to the liver [21]. These results indicate that LJEE attenuated the liver injury in an MCDD-induced NASH animal model.

Fat accumulation in the liver can be recognized as the “first hit” in the pathogenesis of NASH. We found that LJEE produced pharmaceutical effects on hepatic cholesterol and TG levels. Several studies have suggested that hepatic lipogenesis is increased in hepatic steatosis, which may result from either increased TG synthesis, or decreased fatty acid oxidation, both leading to increased TG content in the liver [22]. DGAT2 is a microsomal enzyme that joins acyl-CoA to 1,2-diacylglycerol and thus constitutes the final step in TG biosynthesis [23]. Although MCDD promotes intrahepatic lipid accumulation through impairment of very-low-density lipoprotein (VLDL) secretion [24,25], the mRNA encoding genes related to lipogenesis, such as DGAT2, were suppressed in mice fed a MCDD [26,27], which is in agreement with our findings. Our results showed that DGAT2 expression in MCDD-fed mice was also decreased in the LJEE-treated group. These results demonstrated that LJEE induced down-regulation of lipogenesis-related genes to inhibit accumulation of hepatic lipid droplets via a decrease of TG synthesis in MCDD-fed mice. The microsomal TG transfer protein (MTTP) is responsible for VLDL assembly and export from the liver, which probably contributes to the derangement of triglyceride metabolism [28]. However, reduced MTTP expression has been found in various mouse models of NASH [29,30]. Further direct studies are needed to assess whether LJEE alterations would suffice to correct for the defect. In addition, PPARα mRNA level was significantly increased in LJEE-treated MCDD-fed mice. PPARα plays a central role in the uptake and β-oxidation of fatty acids, especially in the liver, and has been reported to protect against MCDD or high-fat-induced NASH in rodents [31,32]. The beneficial effects of LJEE in the treatment of liver steatosis may be partly due to enhanced fatty acids oxidation in the liver.

Hepatic steatosis increases liver susceptibility to more serious insults like inflammation and increased oxidative stress, especially in the presence of oxidizable unsaturated fatty acids in steatotic livers. The oxidation of unsaturated fatty acids can increase lipid peroxidation, which can activate hepatic stellate cells thus expediting the development of fibrosis [33]. CYP2E1, a member of the oxido-reductase cytochrome family, can oxidize a variety of small molecule substrates including xenobiotics, ethanol, and fatty acids [34]. CYP2E1 expression and activity in the liver are increased in humans and in animal models of NAFLD, which is considered to be the critical “hit” in the transition from benign steatosis to steatohepatitis [34]. In this work, LJEE treatment suppressed CYP2E1 expression, and reduced hepatic MDA level, a marker of lipid peroxidation, in MCDD-fed mice, indicating that treatment with LJEE could abate MCDD-induced oxidative stress in livers.

According to the “two hits theory” for the pathogenesis of NASH, the reactive oxygen products that increase in the “second hit” cause accumulation of lipid peroxidation products, mitochondrial dysfunction, and the increased secretion of pro-inflammatory cytokines such as TNFα [35]. TNFα is an inflammatory cytokine that plays a major role in the progression from steatosis to NASH, and causes secretion of various other cytokines and chemokines [36]. Among these secreted cytokines, the most important one is TGF-β, which plays a pivotal role in hepatic fibrogenesis through hepatic stellate cell (HSC) activation [37]. HSC activation by TNFα and TGF-β results in the expression and deposition of α-SMA and collagen [38]. TNFα also modulates several matrix metalloproteinases that are involved in liver injury, repair, and remodeling, with MMP1 and MMP9 the most relevant MMPs with regards to liver disease [39]. The present study demonstrates that LJEE lowers mRNA levels of hepatic TNFα and TGFβ that are otherwise higher following MCDD feeding. The increased expression of α-SMA and various extracellular matrix-related factors such as collagen I, MMP2, and MMP9 in the livers of MCDD fed mice were also diminished by LJEE treatment. Results from the present research suggest the attenuation of the second hit by LJEE improved liver inflammation and fibrosis induced by the MCDD.

The activation of the regulatory mitogen-activated protein kinase (MAPK) such as JNK and p38, through phosphorylation, usually leads to cell death and inflammation [40,41]. Increased JNK phosphorylation has been reported in NASH model mice, whereas p38 pathway remained unaffected by MCDD [42]. Our data, in full agreement with the published reports [43], demonstrated that MCDD has led to higher phosphorylation of JNK; treatment with LJEE decreased JNK phosphorylation in the NASH model mice. Although inhibition of p38 activation did show a clear association with the benefit effect of LJEE to attenuate hyperglycemia-affected renal dysfunction [15], our data showed that activation of p38 was affected by neither MCDD nor the LJEE administration. In addition, it has been reported that ERK MAPK pathway is not involved in MCDD-induced NASH, however its activation is reported to decrease DGAT2 expression; ERK activation is probably required for the treatment of the disease [42,44]. In this study, we demonstrated that LJEE increased in the extent of ERK phosphorylation, which may lead to increased regeneration of the liver and reduced fat accumulation and inflammation by decreasing the expression of DGAT2, proinflammatory cytokines, and chemokines in NASH. These results suggested that the inhibitory effect of LJEE on the development and progression of NASH is apparently governed by the augmentation of ERK activity and attenuation of JNK activation.

The hepato-protective effects of LJEE against MCDD-induced NASH seems to be effective to those produced by ciprofibrate at the indicated dosage. More than 140 compounds have been isolated and identified from *L. japonica* so far [6]. The specific active constituent(s) of LJEE responsible for its hepato-protective effects remain to be identified in future research.

## 5. Conclusions

Our data suggest that LJEE can prevent hepatic steatosis by reducing hepatic DGAT2 expression, as well as by inducing PPARα expression. Additionally, our data suggest that LJEE inhibits the development of hepatic inflammation and fibrosis by suppressing several inflammatory molecules. The inhibitory effect of LJEE on NASH results from JNK inhibition and increased activation of ERK. LJEE may be an option for NASH prevention and treatment.
